# Two-stage mental health survey of first-line medical staff after ending COVID-19 epidemic assistance and isolation

**DOI:** 10.1007/s00406-021-01239-x

**Published:** 2021-05-18

**Authors:** Li Xu, Dingyun You, Chengyu Li, Xiyu Zhang, Runxu Yang, Chuanyuan Kang, Nianshi Wang, Yuxiong Jin, Jing Yuan, Chao Li, Yujun Wei, Ye Li, Jianzhong Yang

**Affiliations:** 1 Department of Psychiatry, The Second Affiliated Hospital of Kunming Medical University, 374 DianMian Road, Kunming Yunnan, 650101 China; 2 School of Public Health, Kunming Medical University, Kunming Yunnan, 650500 China; 3 School of Health Management, Harbin Medical University, 157 BaoJian Road, Harbin, 150000 Heilongjiang China; 4 Department of Psychiatry, The First Affiliated Hospital of Kunming Medical University, Kunming Yunnan, 650101 China; 5 Department of Psychosomatic Medicine, Shanghai East Hospital, Tongji University School of Medicine, Shanghai, 200120 China

**Keywords:** COVID-19, Medical staff, Mental health

## Abstract

Facing with COVID-19 epidemic such a catastrophic health emergency, the mental health status of medical staff deserves attention. We conducted a two-stage of psychological status monitoring after the end of the assistance and 14 days of isolation, further targeted the vulnerable groups in need of intervention. The study is a cross-sectional survey on 1156 Yunnan medical staff aid to Hubei. Used Cluster sampling method to collect data at 2 time points (at the end of returning from Wuhan and the 14th day of isolation), from March 18, 2020 to April 6, 2020. Female and nurse had higher rates of depressive symptoms than male and doctors and other occupations. The proportion of female with mild and above moderate anxiety levels (22.91%, 2.61%) was higher than male (17.35%, 1.03%) (*p* < 0.05). Female had a better impaired sleep quality (45.06%, 17.49%) more than male (28.57%, 7.94%). Medical staff supported in Wuhan and with junior professional titles reported a higher proportion of sleep quality impairment. At the 14th isolation day stage, the proportion of nurses changed from depression to health (9.15%) and from health to depression (6.1%) better than doctors. The front-line medical staffs had suffered greater psychological pressure in the treatment process of major public health emergency. Researches on the dynamic monitor for the change of psychological status after aiding epidemic areas were still in relatively blank stage. Targeting the vulnerable characteristics of aiding medical staff is significant for effective psychological intervention and sustainable operation of health system.

**Supplementary Information:**

The online version contains supplementary material available at 10.1007/s00406-021-01239-x.

## Introduction

In December 2019, the new coronavirus pneumonia emerged in Wuhan, Hubei Province, and then spread rapidly. This new coronavirus has been officially named [1] ‘SARS COV-2’ by the International Virus Classification Committee, and the disease caused by this virus is called ‘COVID-19’. As the epidemic continued to escalate, the Hubei Provincial Government initiated a level I response to major public health emergencies in the Hubei Province for the prevention and control of COVID-19. Since then, medical teams from all over the country have been dispatching aid to Hubei in batches. As of March 8, 2020, the number of the medical staff from all over the country to assist Wuhan and other parts of Hubei had reached 42,600, fighting side by side with the local medical staff against the epidemic. However, throughout the epidemic response, the shortage of protective equipment, high contagiousness of COVID-19, unknown transmission mechanism and viral characteristics, death of critically ill patients, increased number of infected people in the short-term, media coverage, and many other factors have had a great psychological impact on health care workers [2]. In the performance of their own duty to heal the sick and save the lives of others, the medical staff are also subjected to great threats to their personal safety and psychological stress. It has been previously reported [3, 4] that the mental health of health care workers was affected by the COVID-19 outbreak. Previous studies carried out during severe acute respiratory syndrome (SARS) showed [5] that many health care workers were emotionally affected and traumatized during major infectious outbreaks. One study found that the incidence of depression, insomnia, and post-traumatic stress in nurses during SARS was as high as 38.5%, 37%, and 33%, respectively [6]. Another similar study concluded that during SARS, nurses were under immense psychological stress, and there was a great psychological conflict between their duties as nurses and concerns for their own safety [7]. However, there have been no reports on the mental health status of the medical staff assisting Hubei upon their return from the mission or on the changes in their mental health after 14 days of isolation.

To address this shortcoming, this study quantitatively assessed the psychological status of the Yunnan-aided Hubei medical staff on the first day of their return to Kunming for isolation recuperation. Further, through psychological interventions during the isolation break, such as online psychological counselling sessions, offline reading activities, and group support, the level of depression, anxiety, and insomnia in these medical staff were re-assessed at the end of the 14-day isolation period, and the potential risk factors associated with these symptoms were analyzed. Second, the effectiveness of psychological interventions were measured through longitudinal observation of psychological changes in the medical staff, from a perspective different from that of previous studies, providing an important basis for guiding medical staff to improve their mental health. We hypothesize that the psychological status of the medical staff improved after 14 days of medical isolation compared to the baseline level, and individuals with certain characteristics may have more serious psychological problems.

## Methods

### Research participants

There was a cross-sectional survey based on the Yunnan provincial medical staff aid to Hubei that used a Cluster Sampling method at two time points (days 1 and 14 of isolation). The study lasted from March 18, 2020, to April 6, 2020. During this medical isolation observation period, each member of the medical team lived in a safe epidemic-free hotel with warm care from the provincial government, the original work unit, and the community. Each member of the medical team received psychological adjustment materials provided by the psychological assistance team through the internet. The participants in this survey were all medical staff in Yunnan Province who assisted Wuhan City and Xianning City of Hubei Province. Before the evacuation, all these medical staff members were working in COVID-19-designated hospitals and mobile cabin hospitals in Wuhan and Xianning. There were 1156 individuals in the Yunnan Provincial Medical Assistance Team in the Hubei Province. A total of 1156 questionnaires were both sent out on the 1st day (baseline level) and the 14th day of isolation. A total of 731 participants completed the Patient Health Questionnaire (PHQ-9) and General Anxiety Disorder (GAD-7) questionnaires twice, before and after isolation, with a response rate of 63.23%. A total of 713 participants completed the Pittsburgh Sleep Quality Index (PSQI) scale survey twice, with a response rate of 61.67%.

### Survey methods and questionnaire

Data collection was done through the mobile version of the questionnaire star (www.wjx.cn) applet, where all participants obtained informed consent for the online version before starting the questionnaire. In addition, no approval was required for the study. The informed consent page provided two options (YES/NO); only those who chose “YES” were taken to the questionnaire page, and participants were free to decide to terminate the process. We focused on the symptoms of depression, anxiety, and sleep quality in all participants using the Chinese version of the validated measurement tool [8–11]: the 9-item PHQ-9, 7-item GAD-7, and PSQI [10, 11] scale, respectively. The total scores for these instruments were interpreted as follows: PHQ-9 normal (0–4), mild (5–9), moderate (10–14), severe (15–19), extremely severe (20–27); GAD-7 normal (0–4), mild (5–9), moderate (10–14), severe (15–21); PSQI normal (0–5), mildly impaired (6–10), moderately impaired (11–15), and severely impaired (16–21).

### Demographic information

Basic demographic data included sex (male or female), age (years), occupation [doctor, nurse, other (infection control staff and executive leadership)], marital status [married, unmarried, other (divorced and widowed)], educational background (college/secondary, undergraduate, graduate and above), professional and technical titles certified by the hospital (junior, intermediate, senior), and workplace (Wuhan, non-Wuhan).

### Statistical analysis methods

The statistical analysis for this study was performed using SAS 9.4 software. Participants were included in both the pre-isolation and post-isolation questionnaires as the total number of observed cases, and the counting information was presented in the form of the number of cases (composition ratio). According to the changes in the evaluation types of the participants before and after isolation, they were divided into 4 groups, and the changes in the evaluation types of the Sleep Quality Scale were divided into 3 groups. The *χ*^2^ test was used for inter-group comparisons, and the differences with bilateral *p* < 0.05 were considered statistically significant. The measured information was reduced by the pre-isolation and post-isolation scores on the individual scale, and the difference followed the normal distribution, which was expressed in the form of mean ± standard deviation (*x* ± *s*). The *F* test was used for inter-group comparison. Bilateral values of *p* < 0.05 were considered statistically significant.

## Results

### Analysis of the PHQ-9 test results on the first day of isolation (baseline level)

At the baseline level, women had higher PHQ-9 scores than men, unmarried medical staff had higher PHQ-9 scores than married ones and those with other marital statuses, nurses had higher total PHQ-9 scores than doctors and other staff, and the medical staff members with junior titles had higher PHQ-9 scores than those with intermediate and senior titles (all *p* values < 0.05). Specific demographic data are detailed in Table 1. Through the post test, we found that: medical staff with the characteristics of doctors and nurses, junior and senior, married and unmarried have significant differences in depression (Appendix Table 1).

**Table 1:** Baseline measurement information for the PHQ-9 depression scale

Characteristics	*N* = 731	*x* ± *s*	*F/t*	*p*
Gender				
Male	194	2.59 ± 3.4	20.82*	< .0001
Female	537	3.99 ± 3.77		
Age				
20–35 years	388	3.85 ± 3.74	1.52^§^	0.2184
35–45 years	251	3.37 ± 3.65		
45 years and over	91	3.37 ± 3.83		
Occupation				
Doctor	164	2.84 ± 3.55	5.26^§^	0.0054
Nurse	545	3.88 ± 3.72		
Other	22	3 ± 4.4		
Workplace				
Wuhan	397	3.73 ± 3.85	0.65*	0.4216
Non-Wuhan region	325	3.5 ± 3.57		
Working years				
0–10 years	351	3.75 ± 3.75	0.55^§^	0.5761
10–20 years	232	3.58 ± 3.62		
More than 20 years	148	3.37 ± 3.84		
Job title				
Junior	353	3.94 ± 3.74	4.27^§^	0.0144
Intermediate	246	3.51 ± 3.84		
Senior	121	2.82 ± 3.26		
Marital status				
Married	522	3.44 ± 3.6	3.83^§^	0.0222
Unmarried	184	4.24 ± 4.05		
Other	25	2.76 ± 3.27		
Educational background				
Postgraduate and above	69	2.72 ± 2.96	2.22^§^	0.1097
Bachelor’s degree (undergraduate)	535	3.7 ± 3.86		
College and below	127	3.76 ± 3.48		
Total	1048	3.38 ± 3.59		

*Statistical difference between the two groups was performed by *t* test

^§^Statistical difference among more than two groups was performed by ANOVA

### Analysis of the severity of depression symptoms at baseline

At baseline, the proportion of women experiencing depression (35.38%) was significantly higher than that of men (*p* < 0.05). Women had significantly higher rates of mild (27.37%) and moderate-to-severe (8.01%) depression symptoms than men (17.01%, 4.12%) (*p* < 0.05). The proportion of depression symptoms in nurses was significantly higher (34.87%) than that in doctors (22.56%) and other staff (18.18%) (*p* < 0.05). See Table 2 for details.

**Table 2:** Severity of depression in medical staff at baseline at PHQ-9

Characteristics	*N* = 731	Normal *n* (%)	Mild *n* (%)	Moderate or severe *n* (%)	Z/*χ*^2^	*p*
Gender	731				− 3.6698*	0.0002
Male	194	153 (78.87)	33 (17.01)	8 (4.12)		
Female	537	347 (64.62)	147 (27.37)	43 (8.01)		
Age	730				2.3681^§^	0.306
20–35 years	388	254(65.46)	109 (28.09)	25 (6.44)		
35–45 years	251	180 (71.71)	52 (20.72)	19 (7.57)		
45 years and over	91	65 (71.43)	19 (20.88)	7 (7.69)		
Occupation	731				9.3082^§^	0.0095
Doctor	164	127 (77.44)	27 (16.46)	10 (6.1)		
Nurse	545	355 (65.14)	151 (27.71)	39 (7.16)		
Other	22	18 (81.82)	2 (9.09)	2 (9.09)		
Workplace	722				− 1.0352*	0.3006
Wuhan	397	263 (66.25)	108(27.2)	26 (6.55)		
Non-Wuhan region	325	229 (70.46)	72 (22.15)	24 (7.38)		
Working years	731				1.1628^§^	0.5591
0–10 years	351	233 (66.38)	96 (27.35)	22 (6.27)		
10–20 years	232	160 (68.97)	55 (23.71)	17 (7.33)		
More than 20 years	148	107 (72.3)	29 (19.59)	12 (8.11)		
Job title	720				5.3211^§^	0.0699
Junior	353	228 (64.59)	101 (28.61)	24 (6.8)		
Intermediate	246	172 (69.92)	54 (21.95)	20 (8.13)		
Senior	121	92 (76.03)	23 (19.01)	6 (4.96)		
Marital status	731				5.8083^§^	0.0548
Married	522	369 (70.69)	120 (22.99)	33 (6.32)		
Unmarried	184	113 (61.41)	54 (29.35)	17 (9.24)		
Other	25	18 (72)	6 (24)	1 (4)		
Educational background	731				3.8019^§^	0.1494
Postgraduate and above	69	54 (78.26)	13 (18.84)	2 (2.9)		
Bachelor’s degree (undergraduate)	535	363 (67.85)	128 (23.93)	44 (8.22)		
College and below	127	83 (65.35)	39(30.71)	5 (3.94)		

*And § means statistically significant (*p* < 0.05)

### Transformation of depression symptoms before and after isolation

After a 14-day break in isolation, the emotional state of the medical staff had undergone a certain significant transformation (Table 3, *χ*^2^ = 22.0538). According to Table 3, the proportion of mental health of medical staff before isolation was 70.61%, while that of medical staff after isolation was significantly increased (79.6%) (*χ*^2^ = 22.0538, *p* < 0.0001). At the same time, according to the scores of PHQ-9 scale, the scores after isolation was lower than that before isolation (after isolation: 2.42 ± 3.15, before isolation: 3.38 ± 3.59), the difference was statistically significant. Therefore, it is suggested that the 14-day isolation is helpful to mental health and has a significant positive effect. The data in Table 4 used PHQ-9 scale to collect the mental state before and after isolation. According to the scores, the population can be divided into “health” and “depression”. Therefore, the combination of psychological states before and after isolation has 2 * 2 changes in four psychological states, namely, health to health, health to depression, depression to health and depression to depression.

**Table 3:** Comparison of health and depression of medical staff before and after isolation

	*x* ± *s*	Health	Depression	Total	*χ*^2^	*P*
Time before isolation	3.38 ± 3.59	740 (70.61)	308 (29.39)	1048	22.0538	< .0001
Time after isolation	2.42 ± 3.15	796 (79.6)	204 (20.4)	1000		
Total		1536 (75.0)	512(25.0)	2048

**Table 4:** Transformation of depression before and after isolation of medical staff

Characteristics	*N* = 731	Depression to health *n* (%)	Health to health *n* (%)	Health to depression *n* (%)	Depression to depression *n* (%)	*Z*/*χ*^2^	*p*
Gender	731					− 3.0746*	0.0021
Male	195	18 (9.23)	139 (71.28)	15 (7.69)	23 (11.79)		
Female	536	108 (20.15)	304 (56.72)	42 (7.84)	82 (15.3)		
Age	731					4.4752^§^	0.1067
20–35 years	387	74 (19.12)	220 (56.85)	32 (8.27)	61 (15.76)		
35–45 years	254	39 (15.35)	163 (64.17)	21 (8.27)	31 (12.2)		
45 years and over	90	13 (14.44)	60 (66.67)	4 (4.44)	13 (14.44)		
Occupation	731					7.5186^§^	0.0233
Doctor	164	15 (9.15)	117 (71.34)	10 (6.1)	22(13.41)		
Nurse	545	110 (20.18)	311 (57.06)	44 (8.07)	80(14.68)		
Other	22	1(4.55)	15(68.18)	3 (13.64)	3(13.64)		
Workplace	722					0.9448*	0.3448
Wuhan	397	79 (19.9)	244 (61.46)	19 (4.79)	55(13.85)		
Non-Wuhan region	325	46 (14.15)	194 (59.69)	35 (10.77)	50 (15.38)		
Working years	731					4.9181^§^	0.0855
0–10 years	351	62 (17.66)	206 (58.69)	27(7.69)	56 (15.95)		
10–20 years	232	40 (17.24)	136 (58.62)	24(10.34)	32 (13.79)		
More than 20 years	148	24 (16.22)	101 (68.24)	6 (4.05)	17 (11.49)		
Job title	720					8.1662^§^	0.0169
Junior	353	65 (18.41)	197 (55.81)	31 (8.78)	60 (17)		
Intermediate	246	45 (18.29)	156 (63.41)	16 (6.5)	29 (11.79)		
Senior	121	14 (11.57)	84 (69.42)	8 (6.61)	15 (12.4)		
Marital status	731					7.2468^§^	0.0267
Married	522	81(15.52)	332(63.6)	37(7.09)	72 (13.79)		
Unmarried	184	40(21.74)	95(51.63)	18(9.78)	31 (16.85)		
Other	25	5(0.68)	16(2.19)	2(0.27)	2 (0.27)		
Educational background						1.0962^§^	0.5781
Postgraduate and above	69	5 (7.25)	47 (68.12)	7 (10.14)	10 (14.49)		
Bachelor’s degree (undergraduate)	535	96 (17.94)	323 (60.37)	40 (7.48)	76 (14.21)		
College and below	127	25 (3.42)	73 (9.99)	10 (1.37)	19 (2.6)		

**Fig. 1:**
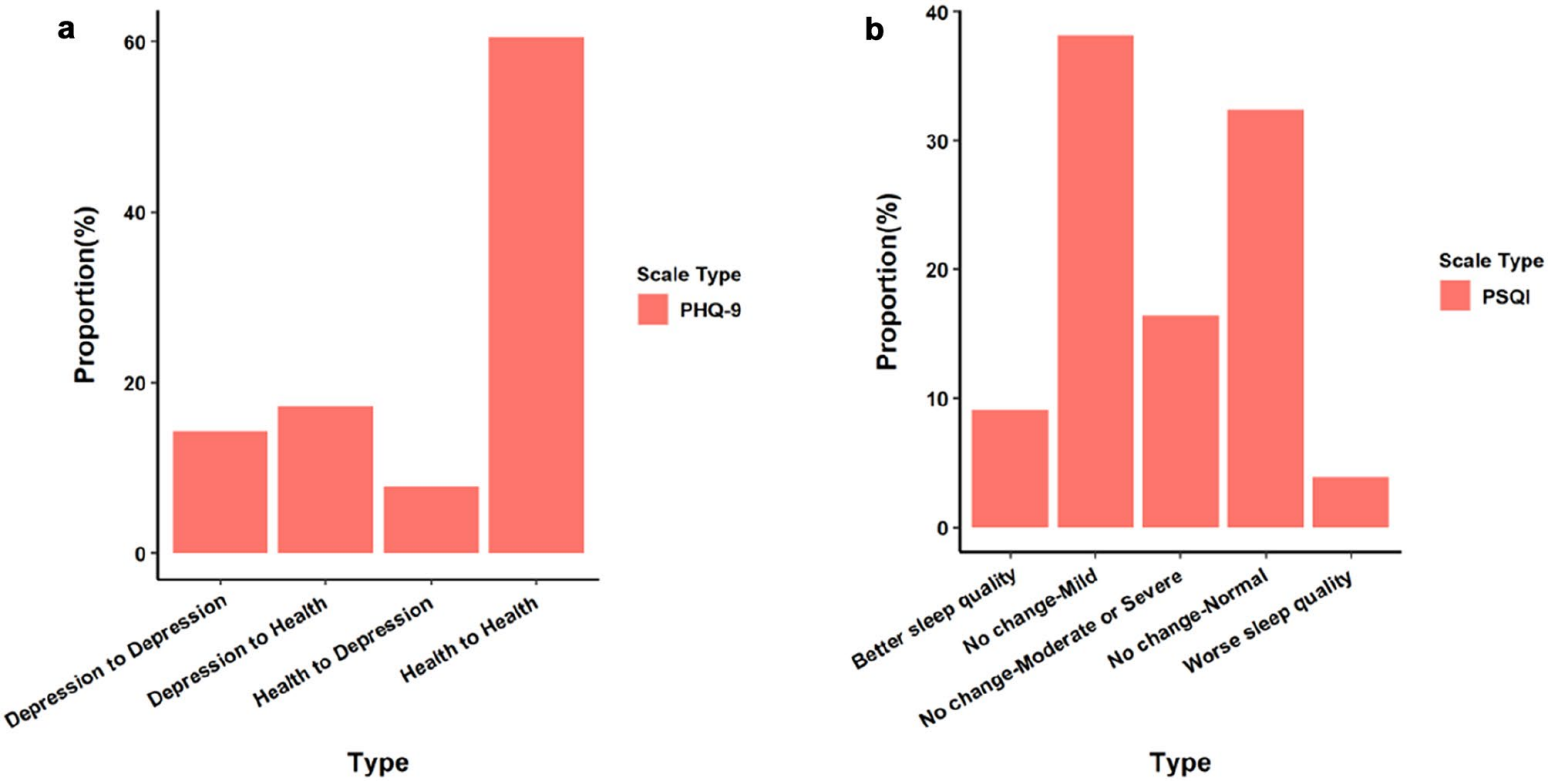
Proportion of changes in depression and quality of sleep before and after isolation

According to Fig. 1a, we can directly observe the proportion of mental state changes and sleep quality changes of medical staff before and after isolation. According to the evaluation results of PHQ-9 scale, the proportion of medical staff whose mental state were healthy was the largest (60.6%), the proportion of medical staff whose mental state changed from depression to health before and after isolation was 17.24%, the medical staff with persistent depression accounted for 14.36%, and those who changed from health to depression accounted for the least (7.8%).

**Fig. 2:**
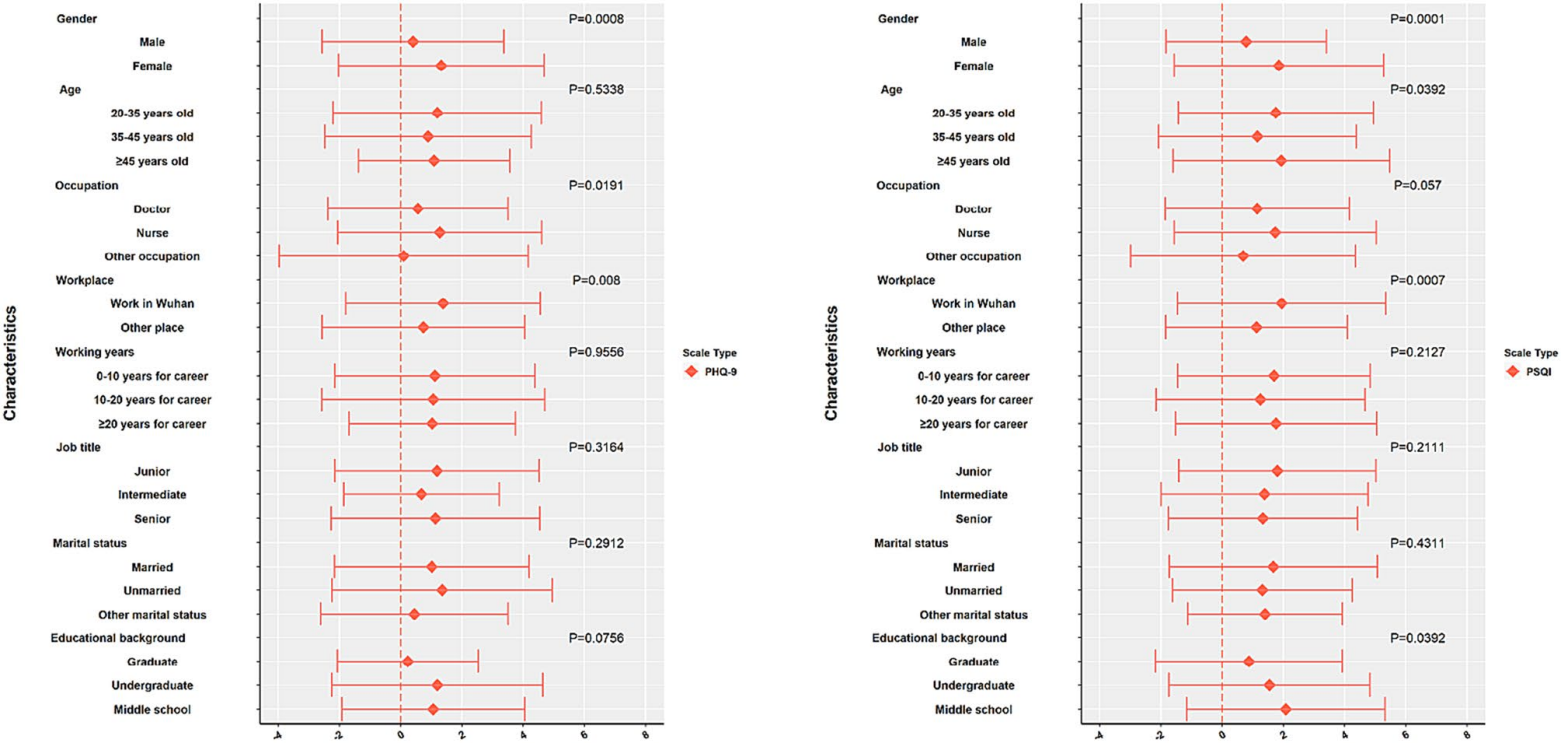
Mean ± SD of the difference between the scores of depression and sleep scale before and after isolation

There were 7.84% of women and 7.69% of men who converted from baseline normal to mild and higher levels of depression. There were 20.15% of women and 9.23% of men who converted from a baseline depression state to normal. We found that 15.3% of women and 11.79% of men were in a constant state of depression both before or after isolation. Approximately 20.18% of nurses transformed from depression to normal health, and the improvement was better than in doctors (9.15%); however, at the same time, the proportion of nurses that changed from normal health to depression (8.07%) was also higher than that in doctors (6.1%). After a series of psychological interventions, the proportion of medical staff working in Wuhan that changed from depression to normal health status was 19.9%, which was significantly better than those in non-Wuhan areas (14.15%). Moreover, the study also found that the situation of the medical staff worked in non-Wuhan areas turning from health to depression after isolation (10.77%) was more severe than those worked in Wuhan areas (4.79%). Up to 8.78% of the medical staff with junior professional titles fell into depression from normal health, a significantly higher proportion than that observed for senior professional titles, as detailed in Table 4. Through further analysis of the above results, it is concluded that: isolation for 14 days is helpful because the proportion of depression to health is higher than that of health to depression, which is statistically different from the results of *Z* value. From the difference ± standard deviation of depression scale score before and after isolation, the same result can be seen (Appendix Table 5). Figure 2 is presented in the form of mean ± standard deviation according to the scores results of PHQ-9 scale and PSQI scale, and the results are consistent with those in Table 4. The specific data can be seen in Appendix Table 5.

### Severity of anxiety during baseline testing for the medical staff

The proportion of women (22.91%, 2.61%) with mild anxiety and above moderate anxiety levels on the first day of isolation (baseline) was higher than that of men (17.35%, 1.03%), (*p* < 0.05). See supplemented Table 1 for details.

### Changes in anxiety levels before and after isolation

Before and after the isolation, some degree of transformation of anxiety occurred in the medical staff, although the difference between before and after isolation was not statistically significant. See supplemental Table 2 for details.

### Analysis of impaired sleep quality of the medical staff at baseline

There were 715 participants who completed the PSQI scale test before isolation. On the first day of isolation (baseline), women had a higher proportion of mildly impaired and moderately impaired sleep quality (45.06%, 17.49%, respectively) compared to men (28.57%, 7.94%, respectively), whereas 63.49% of men reported normal sleep quality, much higher than the 37.45% reported for women. A higher proportion of doctors reported normal sleep quality (60.74%) than nurses (39.43%) and other staff (40.91%). Compared to the medical staff members who were not in the Wuhan area, those worked in Wuhan reported a higher proportion of mild or moderate and severe sleep quality impairment. Nearly 61% of the staff with junior professional titles experienced mild and moderate sleep impairments, higher than the proportion reported in staff with intermediate and senior titles. The medical staff members whose marital status was ‘married’ (45.49%) reported a higher proportion of normal sleep quality than those who were unmarried (42.22%) and of other marital statuses (36%). Educational background at the college level and below manifested as a higher proportion of mild or moderately moderate impairment of sleep quality (all *p* < 0.05). See Table 5 for details.

**Table 5:** Baseline analysis of sleep quality impairment in PSQI

Characteristics	*N* = 715	Normal *n* (%)	Mild *n* (%)	Moderate or severe *n* (%)	Z*/χ*^2^	*p*
Gender	715				− 6.1073*	< .0001
Male	189	120 (63.49)	54 (28.57)	15 (7.94)		
Female	526	197 (37.45)	237 (45.06)	92 (17.49)		
Age	714				3.4172^§^	0.1811
20–35 years	376	152 (40.43)	168 (44.68)	56 (14.89)		
35–45 years	247	122 (49.39)	90 (36.44)	35 (14.17)		
45 years and over	91	42 (46.15)	33 (36.26)	16 (17.58)		
Occupation	715				21.2316^§^	< .0001
Doctor	163	99 (60.74)	48 (29.45)	16 (9.82)		
Nurse	530	209 (39.43)	234 (44.15)	87 (16.42)		
Other	22	9 (40.91)	9 (40.91)	4 (18.18)		
Workplace	707				-3.2879*	0.001
Wuhan	388	151 (38.92)	172 (44.33)	65 (16.75)		
Non-Wuhan region	319	164 (51.41)	116 (36.36)	39 (12.23)		
Working years	715				0.0767^§^	0.9624
0–10 years	342	146 (42.69)	152 (44.44)	44 (12.87)		
10–20 years	225	101 (44.89)	87 (38.67)	37 (16.44)		
More than 20 years	148	70 (47.3)	52 (35.14)	26 (17.57)		
Job title	705				9.6394^§^	0.0081
Junior	343	134 (39.07)	151 (44.02)	58 (16.91)		
Intermediate	241	114(47.3)	92(38.17)	35 (14.52)		
Senior	121	66 (54.55)	42 (34.71)	13 (10.74)		
Marital status	715				1.9136^§^	0.3841
Married	510	232 (45.49)	196 (38.43)	82 (16.08)		
Unmarried	180	76 (42.22)	86 (47.78)	18 (10)		
Other	25	9 (36)	9 (36)	7 (28)		
Educational background	715				11.1597^§^	0.0038
Postgraduate and above	69	41 (59.42)	22 (31.88)	6 (8.7)		
Bachelor’s degree (undergraduate)	523	232 (44.36)	215 (41.11)	76 (14.53)		
College and below	123	44 (35.77)	54 (43.9)	25 (20.33)		

### Analysis of changes in the quality of sleep of the medical staff before and after isolation

There were 713 participants who completed the two PSQI scale tests, showing the following results: a significant change in sleep quality of medical staff before and after isolation was reported (Table 6, *χ*^2^ = 28.8414). Similarly, the improvement of sleep quality can also be observed in the 14-day isolation. The proportion of medical staff with good sleep quality after isolation was 7.17% more than that before isolation (*χ*^2^ = 28.8414, *p* < 0.0001). According to the scores of PSQI scale, the scores after isolation was also lower than that before isolation (after isolation: 4.72 ± 3.26, before isolation: 6.23 ± 3.74), the difference was statistically significant (Table 6).

**Table 6:** Comparison of quality of sleep of medical staff before and after isolation

	*x* ± *s*	Good quality of sleep	Poor quality of sleep	Total	*χ*^2^	*P*
Time before isolation	6.23 ± 3.74	890 (86.49)	139 (13.51)	1029	28.8414	< .0001
Time after isolation	4.72 ± 3.26	930 (93.66)	63 (6.34)	993		
Total		1820 (90.00)	202 (9.99)	2022		

According to Fig. 1b, According to the evaluation results of PSQI scale, the proportion of medical staff whose sleep quality remained normal before and after isolation was the largest (38.15%), followed by the medical staff with good sleep quality (32.4%) and the medical staff with moderate or severe poor sleep quality (16.41%). It can be seen that the sleep quality of most medical staff remains unchanged before and after isolation. Different from mental state, before and after isolation, the proportion of medical staff who got better sleep quality was 9.12%, and those who became worse accounted for the least (3.93%).

A higher proportion of women (36.9%) reported better sleep quality than men (20.0%). At the test endpoint, a higher proportion of women (22.57%) had mild, moderate, and more impaired sleep quality. The medical staff members in the 20–35-year age group obtained better sleep after 14-day isolation. Compared to doctors (22.22%) and other medical staff (27.27%), the sleep quality of more nurses had improved (35.73%). After 14 days of rest adjustment, the medical staff that once worked in Wuhan had a large proportion of sleep improvement, whereas the medical staff worked in non-Wuhan areas had a larger proportion of sleep deterioration. See Table 7 for details. Figure 2 is presented in the form of mean ± standard deviation according to the scores results of PHQ-9 scale and PSQI scale, and the results are consistent with those in Table 5. The specific data can be seen in Appendix Table 6.

**Table 7:** Analysis of changes in sleep quality before and after isolation

Characteristics	*N* = 713	Better sleep quality *n* (%)	No change in sleep quality (*n* = 417)			Worse sleep quality *n* (%)	Z/*χ*^2^	*P*
			Normal *n* (%)	Mild *n* (%)	Moderate or severe *n *(%)			
Gender	713						1.6596*	0.097
Male	190	38 (20)	107 (56.32)	20 (10.53)	7 (3.68)	18 (9.47)		
Female	523	193 (36.9)	165 (31.55)	97 (18.55)	21 (4.02)	47 (8.99)		
Age	713						5.4957^§^	0.0641
20–35 years	374	140 (37.43)	127 (33.96)	65 (17.38)	9 (2.41)	33 (8.82)		
35–45 years	249	63 (25.3)	110 (44.18)	39 (15.66)	13 (5.22)	24 (9.64)		
45 years and over	90	28 (31.11)	35 (38.89)	13 (14.44)	6 (6.67)	8 (8.89)		
Occupation	713						2.617^§^	0.2702
Doctor	162	36 (22.22)	87 (53.7)	18 (11.11)	7 (4.32)	14 (8.64)		
Nurse	529	189 (35.73)	178 (33.65)	95 (17.96)	19 (3.59)	48 (9.07)		
Other	22	6 (27.27)	7 (31.82)	4 (18.18)	2 (9.09)	3 (13.64)		
Workplace	705						2.7737*	0.0055
Wuhan	388	146 (37.63)	134 (34.54)	67 (17.27)	14 (3.61)	27 (6.96)		
Non-Wuhan region	317	83 (26.18)	136 (42.9)	47 (14.83)	13 (4.1)	38 (11.99)		
Working years	713						3.5631^§^	0.1684
0–10 years	341	123 (36.07)	122 (35.78)	60 (17.6)	7 (2.05)	29 (8.5)		
10–20 years	224	65 (29.02)	87 (38.84)	35 (15.63)	12 (5.36)	25 (11.16)		
More than 20 years	148	43 (29.05)	63 (42.57)	22 (14.86)	9 (6.08)	11 (7.43)		
Job title	703						1.3318^§^	0.5138
Junior	343	126 (36.73)	113 (32.94)	61 (17.78)	11 (3.21)	32 (9.33)		
Intermediate	239	68 (28.45)	102 (42.68)	39 (16.32)	12 (5.02)	18 (7.53)		
Senior	121	34 (28.1)	55 (45.45)	14 (11.57)	5 (4.13)	13 (10.74)		
Marital status	713						0.4634^§^	0.7932
Married	508	164 (32.28)	202 (39.76)	77 (15.16)	22 (4.33)	43 (8.46)		
Unmarried	180	59 (32.78)	61 (33.89)	35 (19.44)	4 (2.22)	21 (11.67)		
Other	25	8 (32)	9 (36)	5 (20)	2 (8)	1 (4)		
Educational background	713						3.039^§^	0.2188
Postgraduate and above	69	18 (26.09)	34 (49.28)	6 (8.7)	3 (4.35)	8 (11.59)		
Bachelor’s degree (undergraduate)	521	163 (31.29)	199 (38.2)	89 (17.08)	21 (4.03)	49 (9.4)		
College and below	123	50 (40.65)	39 (31.71)	22 (17.89)	4 (3.25)	8 (6.5)		

## Discussion

Our research findings indicated that after completing the epidemic assistance task in Hubei, the overall levels of anxiety, depression, and sleep impairment were relatively high. Among them, women, nurses, and medical staff with junior professional titles were more vulnerable to psychological and sleep problems. After 14 days of psychological intervention adjustment, women and nurses had become the main beneficiaries of psychological and sleep improvement. In addition, medical staff aged 20–35 years were also a group that experienced sleep improvement. For the medical staff who worked in the non-Wuhan areas, their mental health and sleep state worsened more than those who worked in the Wuhan area.

Before and after medical isolation, the proportion of improvement in the psychological status of the medical staff was much higher than the proportion of deterioration. This may have something to do with being far away from the front line of the epidemic and the development of the epidemic. Keeping away from possibly infected individuals relieves the medical staff of the pressure of a safety threat, which improves the psychological status [12]. In addition, with the appropriate intervention of the government, the epidemic situation had gradually been controlled, which could also be one of the reasons for the psychological relief of the medical staff before and after isolation.

### High level of depression, anxiety, and sleep quality impairment

We investigated 731 medical staff that supported the COVID-19 epidemic. Although each medical unit dispatched medical personnel with professional and psychological qualities to participate in the work of assisting Hubei to fight the epidemic, at the end of assistance work, 31.6%, 23.6%, and 55.6% of the participants still showed symptoms of depression, anxiety, and impaired sleep quality, respectively. Our results are largely consistent with those of other studies, which have reported that during a major public health emergency, the medical staff faced the risk of experiencing serious mental health consequences due to high-intensity fatigue [13–15]. As a COVID-19 confrontation, research evidence demonstrated that 50.4%, 44.6%, and 34.0% of the medical staff experienced depression, anxiety, and insomnia, respectively [4], showing a worse mental health status than is shown by our research. There are two main reasons for the lower levels of depression and anxiety of our survey participants. First, the respondents entered the epidemic area at the controlled stage of the epidemic in Hubei, and the Chinese experts had figured out the method of transmission of the coronavirus and formulated adequate treatment plans for patients. Second, the medical staff had returned to a safe and comfortable environment, where they no longer needed to face highly infectious patients and carry out high-intensity isolation treatment. These two factors may have contributed to the reduction of the rates of depression and anxiety compared to those experienced during the peak period of the epidemic. However, it should be noted that the proportion of sleep quality impairment increased, which did not rule out insomnia caused by the post-traumatic stress response. After prolonged close contact with critically ill patients, the medical staff worried that they would be infected and transmit the infection to family and friends [16], causing them to experience sleep quality impairment.

### Focusing on the characteristics of vulnerable groups after the epidemic assistance

Our research found that women and nurses were more predisposed to depression, anxiety, and sleep impairment than men and doctors. This conclusion had also been arrived at in other studies [17–22], where the incidence of stress-related mental illness was twice as high in women than in men. First, as common family caregivers, women are more prone to worry about their family’s health and well-being. Female medical staff worry that their families are not cared for, which leads to psychological problems and the inability to work at ease [23]. Second, according to a working paper by the NATIONAL BUREAU OF ECONOMIC RESEARCH (NBER) in June 2020 (working paper 27,359), women’s perception of COVID-19 may also cause a gender gap. Due to the fact that women’s perception of the severity of COVID-19 is more prominent than that of men, the resulting panic and pressure are also more obvious, which is one of the reasons for the frequent psychological problems among female medical staff. Moreover, in the fight against such a catastrophic public health emergency, nurses assumed more daily care for patients and spent more time in the isolation ward with critical-ill patients. The nursing services they provided included not only the treatment of the disease and administration of drugs, but also the counselling of patients’ negative psychological emotions [6]. All this excess workload pushed nurses to face increasing mental stress. At the same time, a large number of nurses were young, with only a few years of work experience. Insufficient nursing experience in responding to such pandemics had caused them to be more prone to psychological problems and sleep impairment.

### Effect evaluation after 14-day isolation of psychological intervention

**Fig. 3:**
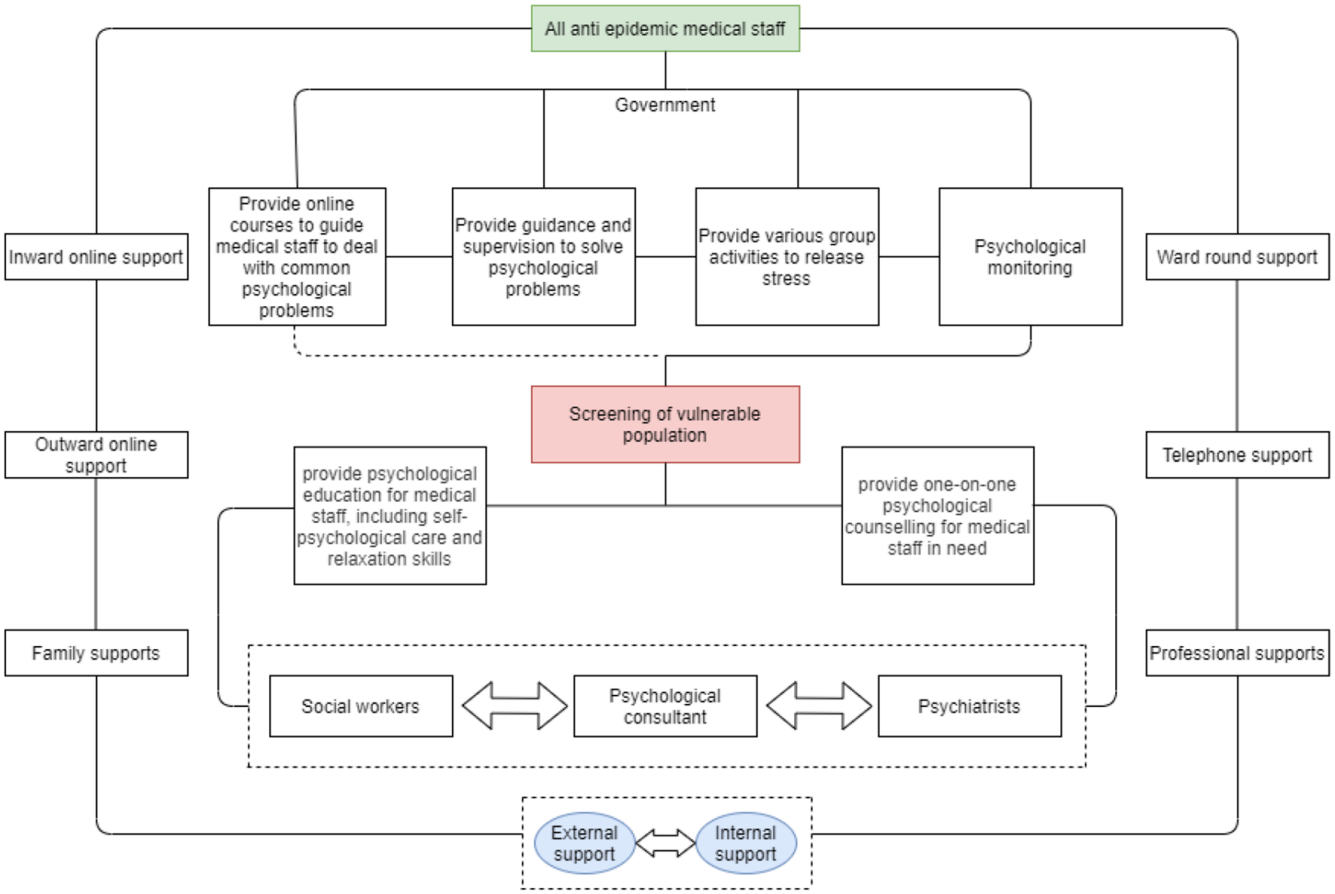
Psychological intervention process of anti-epidemic medical staff

For further evaluation of the psychological intervention and targeting of groups that continue to be vulnerable, we found that after 14 days of medical isolation, observation, and psychological adjustment, the emotional status of the medical staff changed. At the end of the second investigation, 22.16%, 13.67%, and 29.45% of the medical staff were still in a state of depression, anxiety, and sleep quality impairment, respectively. However, compared with the level before isolation and intervention, it showed a significant decline. As revealed in other surveys, adequate psychological training, social support of superiors, and communication could have a positive impact on mental health [24–26]. During the 14 days of isolation, the combination of online psychological counselling, online group psychological activities, unit leaders’ condolences, encouragement, reading, and other intervention activities could support the reduction of the psychological pressure of the medical staff. As shown in Fig. 3, the psychological intervention for anti-epidemic medical staff is carried out with the participation of multiple subjects at different levels. The government is responsible for organizing a series of activities such as providing online courses to help deal with common psychological problems, providing guidance and supervision to solve psychological problems, providing different group activities to release pressure [27], and 7 * 24 psychological state assessments. After the screening of characteristics of vulnerable population, psychological consultant and psychiatrist provide professional psychological education, relaxation skills and one-on-one counselling by telephone and video connection. It is worth mentioning that some areas follow an online social psychological support model [28] integrating family members, social workers, psychologists and psychiatrists to provide social support for anti-epidemic medical staff. In addition, the general anxiety disorder-7 (GAD-7), mood index questionnaire, Pittsburgh sleep quality index and other tools were used to screen out the medical staff with the characteristics of psychological vulnerability, and carry out psychological education and one-to-one psychological counselling services for them [29]. In this process, the inspired, plan and deter (APD) responder risk and resilience model is applied. At present, the above model has been developed and implemented by West China Hospital, integrating online and offline, early intervention and rehabilitation. And according to the model adopted by psychological counselling, stress response and emotion should be focused on during the early stage of the epidemic, and internalized emotion and physical symptoms should be focused on during the middle and late stages of the epidemic. Especially for women and nurses, the above measures were very beneficial for the recovery of their mental state, and the transformation of the proportion of depression to healthy status was greatly increased. However, we still found vulnerable groups with mental and sleep deterioration after isolation and psychological intervention. The susceptible subgroups of medical staff were mostly the medical staff with junior professional titles and medical staff worked in the non-Wuhan areas during the epidemic assistance. Insufficient experience and psychological adjustment mechanisms for major public health emergencies caused these medical staff with little work experience to be easily trapped in the dilemma of post-stress trauma. Medical staff with fewer years of work experience in responding to public health emergencies often exhibit a poorer mental state, resilience, and social support; as such, they are more likely to experience psychological distress. Previous studies on avian influenza A/H7N9 have shown that medical staff with < 5 years of work experience or without relevant training and experience are more likely to have psychological problems [30]. Nevertheless, the long-standing medical staff have extensive experience, including previous experience in public health emergencies, such as SARS and H1N1/swine flu. Therefore, compared to new employees, they know how to better protect themselves and have the confidence to beat the epidemic, which is helpful to enhance their mental health [31, 32]. All the evidence reminds us of more precise and multi-dimensional protective psychological interventions that should be adopted for medical staff supporting the epidemic and the fact that constant attention should be paid to their mental health recovery.

### Limitations

However, this study has several limitations. The first limitation was the range of participants, all of whom were from the Yunnan Province, which limits the extension of our findings to other regions. Second, this study was an online survey, and the findings were self-reported; therefore, there was a lack of adequate access to face-to-face interviews, and some important information may have been missed. Third, this study was unable to distinguish between the participants’ own pre-existing mental health symptoms and symptoms that emerged during the epidemic. Fourth, the response rate of the questionnaire in this study was 63.23%. However, we cannot exclude the possibility that some of the cases of non-response were due to the inability of the respondents to provide answers due to mental stress, or that there was no stress at all, and they were not interested in this survey; in such cases, response bias may still exist.

### Generalisability

With the COVID-19 global pandemic and its significant burden on the medical staff, our study provides evidence for benefits of psychological interventions by targeting two stages of mental health change in the medical staff and targeting key intervention populations. However, all participants were from the Yunnan Province, which may prevent the extension of these findings to other regions.

## Supplementary Information

Below is the link to the electronic supplementary material.

Supplementary file1 (DOC 220 KB)
