# Supplementary material for: Two-stage mental health survey of first-line medical staff after ending COVID-19 epidemic assistance and isolation
Source: Eur Arch Psychiatry Clin Neurosci. 2021 May 18;272(1):81–93. doi: 10.1007/s00406-021-01239-x (PMC8130787; doi:10.1007/s00406-021-01239-x)
Supplement: Supplementary file 1 — Supplementary file1 (DOC 220 KB) [file 406_2021_1239_MOESM1_ESM.doc]

**Appendix**

| Appendix Table 1. post hoc test for characteristics with statistcal significant difference | | | | |
| --- | --- | --- | --- | --- |
| **Characteristics** | **x-x'** | **95%CI** | ***F/t*** | ***p*** |
| **Occupation** |  |  | 5.26 | 0.0054 |
| Nurse-Other | 0.8789 | ((-0.7023)-2.4601) |  |  |
| Nurse-Doctor*** | 1.0374 | (0.3898-1.685) |  |  |
| Other-Nurse | -0.8789 | ((-2.4601)-0.7023) |  |  |
| Other-Doctor | 0.1585 | ((-1.4924)-1.8094) |  |  |
| Doctor-Nurse*** | -1.0374 | ((-1.685)-(-0.3898)) |  |  |
| Doctor-Other | -0.1585 | ((-1.8094)-1.4924) |  |  |
| **Job title** |  |  | 4.27 | 0.0144 |
| Junior-Intermediate | 0.4283 | ((-0.1747)-1.0313) |  |  |
| Junior-Senior*** | 1.1223 | (0.3575-1.8871) |  |  |
| Intermediate-Junior | -0.4283 | ((-1.0313)-0.1747) |  |  |
| Intermediate-Senior | 0.694 | ((-0.1122)-1.5002) |  |  |
| Senior-Junior*** | -1.1223 | ((-1.8871)-(-0.3575)) |  |  |
| Senior-Intermediate | -0.694 | ((-1.5002)-0.1122) |  |  |
| **Marital status** |  |  | 3.83 | 0.0222 |
| Unmarried-Married*** | 0.7966 | (0.172-1.4212) |  |  |
| Unmarried-Other | 1.4791 | ((-0.0737)-3.032) |  |  |
| Married-Unmarried*** | -0.7966 | ((-1.4212)-(-0.172)) |  |  |
| Married-Other | 0.6825 | ((-0.809)-2.1741) |  |  |
| Other-Unmarried | -1.4791 | ((-3.032)-0.0737) |  |  |
| Other-Married | -0.6825 | ((-2.1741)-0.809) |  |  |
| **Comparisons significant at the 0.05 level are indicated by ***.** |  |  |  |  |

| Appendix Table 2. Wilcoxon Two-Sample Test for characteristics with statistcal significant difference | | | | | |
| --- | --- | --- | --- | --- | --- |
| **Characteristics** | | | Z/ꭓ^2^ | ***p*** | |
| **Occupation** | | | 9.3082^§^ | 0.0095 | |
| Doctor-Nurse | | | -2.7997 | 0.0051 | |
| Doctor-Other | | | -0.3589 | 0.7197 | |
| Nurse-Other | | | -0.0133 | 0.9894 | |
| **Comparisons significant at the 0.05 level are indicated by ***.** | | |  |  | |
| Appendix Table 3. Wilcoxon Two-Sample Test for characteristics with statistcal significant difference | | | | |  |
| **Characteristics** | **Z/ꭓ2** | ***p*** | | |  |
| **Occupation** | 7.5186^§^ | 0.0233 | | |  |
| Doctor-Nurse | -2.7068 | 0.0068 | | |  |
| Doctor-Other | 0.3561 | 0.7218 | | |  |
| Nurse-Other | -0.6713 | 0.502 | | |  |
| **Job title** | 8.1662^§^ | 0.0169 | | |  |
| Junior-Intermediate | -2.1217 | 0.0339 | | |  |
| Intermediate-Senior | -0.8509 | 0.3948 | | |  |
| Senior-Junior | -2.4378 | 0.0148 | | |  |
| **Marital status** | 7.2468^§^ | 0.0267 | | |  |
| Married-Unmarried | 2.6243 | 0.0087 | | |  |
| Unmarried-Other | -1.3092 | 0.1905 | | |  |
| Married-Other | -0.2582 | 0.7962 | | |  |
| **Comparisons significant at the 0.05 level are indicated by ***.** |  |  | | |  |

| Appendix Table 4. Wilcoxon Two-Sample Test for characteristics with statistcal significant difference | | |
| --- | --- | --- |
| **Characteristics** | **Z/ꭓ2** | ***P*** |
| **Occupation** | 21.2316^§^ | <.0001 |
| Doctor-Nurse | -4.5925 | <.0001 |
| Doctor-Other | 1.8313 | 0.0671 |
| Nurse-Other | -0.0133 | 0.9894 |
| **Job title** | 9.6394^§^ | 0.0081 |
| Junior-Intermediate | -1.8556 | 0.0635 |
| Intermediate-Senior | -1.3987 | 0.1619 |
| Senior-Junior | -2.9589 | 0.0031 |
| **Educational background** | 11.1597^§^ | 0.0038 |
| Graduate-Undergraduate | -2.3829 | 0.0172 |
| Undergraduate-Middle School | 1.9831 | 0.0474 |
| Graduate-Middle School | -3.2821 | 0.001 |
| **Comparisons significant at the 0.05 level are indicated by ***.** |  |  |

|  | Appendix Table 5. Difference of PHQ-9 scores before and after isolation | | | | | | | | | | | | | | |  | | |
| --- | --- | --- | --- | --- | --- | --- | --- | --- | --- | --- | --- | --- | --- | --- | --- | --- | --- | --- |
|  | **Characteristics** | | | **N=731** | | **x±s** | | ***F/t*** | | | | ***P*** | | | |  | | |
|  |  | | |
|  | **Gender** | | |  | |  | | 11.38* | | | | 0.0008 | | | |  | | |
|  | Male | | | 195 | | 0.4±2.97 | |  | | | |  | | | |  | | |
|  | Female | | | 536 | | 1.32±3.36 | |  | | | |  | | | |  | | |
|  | **Age** | | |  | |  | | 0.63^§^ | | | | 0.5338 | | | |  | | |
|  | 20-35 years | | | 387 | | 1.19±3.40 | |  | | | |  | | | |  | | |
|  | 35-45 years | | | 254 | | 0.89±3.37 | |  | | | |  | | | |  | | |
|  | 45 years and over | | | 90 | | 1.089±2.47 | |  | | | |  | | | |  | | |
|  | **Occupation** | | |  | |  | | 3.98^§^ | | | | 0.0191 | | | |  | | |
|  | Doctor | | | 164 | | 0.56±2.94 | |  | | | |  | | | |  | | |
|  | Nurse | | | 545 | | 1.27±3.33 | |  | | | |  | | | |  | | |
|  | Other | | | 22 | | 0.09±4.07 | |  | | | |  | | | |  | | |
|  | **Workplace** | | |  | |  | | 7.08* | | | | 0.008 | | | |  | | |
|  | Wuhan | | | 397 | | 1.38±3.18 | |  | | | |  | | | |  | | |
|  | Non-Wuhan region | | | 325 | | 0.74±3.31 | |  | | | |  | | | |  | | |
|  | **Working years** | | |  | |  | | 0.05^§^ | | | | 0.9556 | | | |  | | |
|  | 0-10 years | | | 351 | | 1.11±3.27 | |  | | | |  | | | |  | | |
|  | 10-20 years | | | 232 | | 1.06±3.64 | |  | | | |  | | | |  | | |
|  | More than 20 years | | | 148 | | 1.02±2.72 | |  | | | |  | | | |  | | |
|  | **Job title** | | |  | |  | | 1.15^§^ | | | | 0.3164 | | | |  | | |
|  | Junior | | | 353 | | 1.18±3.34 | |  | | | |  | | | |  | | |
|  | Intermediate | | | 121 | | 0.67±2.54 | |  | | | |  | | | |  | | |
|  | senior | | | 246 | | 1.13±3.41 | |  | | | |  | | | |  | | |
|  | **Marital status** | | |  | |  | | 1.24^§^ | | | | 0.2912 | | | |  | | |
|  | married | | | 522 | | 1.01±3.18 | |  | | | |  | | | |  | | |
|  | unmarried | | | 184 | | 1.35±3.6 | |  | | | |  | | | |  | | |
|  | other | | | 25 | | 0.44±3.06 | |  | | | |  | | | |  | | |
|  | **Educational background** | | |  | |  | | 2.59^§^ | | | | 0.0756 | | | |  | | |
|  | Postgraduate and above | | | 69 | | 0.23±2.3 | |  | | | |  | | | |  | | |
|  | Bachelor's degree (undergraduate) | | | 535 | | 1.19±3.45 | |  | | | |  | | | |  | | |
|  | College and below | | | 127 | | 1.06±2.99 | |  | | | |  | | | |  | | |
|  | **Total** | | | 920 | | 1.06±3.15 | |  | | | |  | | | |  | | |
|  | * Statistical difference between two groups was performed by T test. | | |  | |  | |  | | | |  | | | |  | | |
|  | § Statistical difference among more than two groups was performed by ANOVA. | | |  | |  | |  | | | |  | | | |  | | |
|  | Appendix Table 6. Difference of quality of PSQI scores before and after isolation | | | | | | | | | | | | | | | | | |
|  | **Characteristics** | | | | | | **N=713** | | **x±s** | | ***F/t*** | | | ***P*** | | | | |
|  |
|  | **Gender** | | | | | |  | |  | | 15.16* | | | 0.0001 | | | | |
|  | Male | | | | | | 195 | | 0.78±2.62 | |  | | |  | | | | |
|  | Female | | | | | | 536 | | 1.85±3.42 | |  | | |  | | | | |
|  | **Age** | | | | | |  | |  | | 3.25^§^ | | | 0.0392 | | | | |
|  | 20-35 years | | | | | | 387 | | 1.75±3.19 | |  | | |  | | | | |
|  | 35-45 years | | | | | | 254 | | 1.15±3.23 | |  | | |  | | | | |
|  | 45 years and over | | | | | | 90 | | 1.93±3.54 | |  | | |  | | | | |
|  | **Occupation** | | | | | |  | |  | | 2.88^§^ | | | 0.057 | | | | |
|  | Doctor | | | | | | 164 | | 1.14±3.01 | |  | | |  | | | | |
|  | Nurse | | | | | | 545 | | 1.73±3.3 | |  | | |  | | | | |
|  | Other | | | | | | 22 | | 0.68±3.67 | |  | | |  | | | | |
|  | **Workplace** | | | | | |  | |  | | 11.55* | | | 0.0007 | | | | |
|  | Wuhan | | | | | | 397 | | 1.94±3.4 | |  | | |  | | | | |
|  | Non-Wuhan region | | | | | | 325 | | 1.12±2.97 | |  | | |  | | | | |
|  | **Working years** | | | | | |  | |  | | 1.55^§^ | | | 0.2127 | | | | |
|  | 0-10 years | | | | | | 351 | | 1.69±3.14 | |  | | |  | | | | |
|  | 10-20 years | | | | | | 232 | | 1.25±3.41 | |  | | |  | | | | |
|  | More than 20 years | | | | | | 148 | | 1.76±3.29 | |  | | |  | | | | |
|  | **Job title** | | | | | |  | |  | | 1.56^§^ | | | 0.2111 | | | | |
|  | Junior | | | | | | 353 | | 1.8±3.22 | |  | | |  | | | | |
|  | Intermediate | | | | | | 246 | | 1.38±3.38 | |  | | |  | | | | |
|  | senior | | | | | | 121 | | 1.33±3.09 | |  | | |  | | | | |
|  | **Marital status** | | | | | |  | |  | | 0.84^§^ | | | 0.4311 | | | | |
|  | married | | | | | | 522 | | 1.67±3.4 | |  | | |  | | | | |
|  | unmarried | | | | | | 184 | | 1.31±2.93 | |  | | |  | | | | |
|  | other | | | | | | 25 | | 1.4±2.52 | |  | | |  | | | | |
|  | **Educational background** | | | | | |  | |  | | 3.15^§^ | | | 0.0433 | | | | |
|  | Postgraduate and above | | | | | | 69 | | 0.87±3.05 | |  | | |  | | | | |
|  | Bachelor's degree (undergraduate) | | | | | | 535 | | 1.54±3.28 | |  | | |  | | | | |
|  | College and below | | | | | | 127 | | 2.08±3.24 | |  | | |  | | | | |
|  | **Total** | | | | | | 898 | | 1.6±3.22 | |  | | |  | | | | |
|  | * Statistical difference between two groups was performed by T test. | | | | | |  | |  | |  | | |  | | | | |
|  | § Statistical difference among more than two groups was performed by ANOVA. | | | | | | | | | | | | |  |  | |  |  |
| Appendix Table 7. Assessment for selectivity of data between participants and non participants | | | | | | | | | | | | | | | | | | |
| **Characteristics** | | **N=1128** | **Non participants n(%)** | | **Participants n(%)** | | | | | χ^2^ | | | ***P*** | | | | | |
| **Gender** | |  |  | |  | | | | | 1.3463 | | | 0.2459 | | | | | |
| Male | | 331 | 101(30.51) | | 230(69.49) | | | | |  | | |  | | | | | |
| Female | | 797 | 216(27.1) | | 581(72.9) | | | | |  | | |  | | | | | |
| **Age** | |  |  | |  | | | | | 2.5353 | | | 0.2815 | | | | | |
| 20-35 years | | 608 | 181(29.77) | | 427(70.23) | | | | |  | | |  | | | | | |
| 35-45 years | | 379 | 103(27.18) | | 276(72.82) | | | | |  | | |  | | | | | |
| 45 years and over | | 141 | 33(23.4) | | 108(76.6) | | | | |  | | |  | | | | | |
| **Occupation** | |  |  | |  | | | | | 2.6801 | | | 0.2618 | | | | | |
| Doctor | | 250 | 86(34.4) | | 164(65.6) | | | | |  | | |  | | | | | |
| Nurse | | 767 | 222(28.94) | | 545(71.06) | | | | |  | | |  | | | | | |
| Other | | 31 | 9(29.03) | | 22(70.97) | | | | |  | | |  | | | | | |
| **Workplace** | |  |  | |  | | | | | 6.1083 | | | 0.0135 | | | | | |
| Wuhan | | 595 | 198(33.28) | | 397(66.72) | | | | |  | | |  | | | | | |
| Non-Wuhan region | | 440 | 115(26.14) | | 325(73.86) | | | | |  | | |  | | | | | |
| **Working years** | |  |  | |  | | | | | 158.9886 | | | <.0001 | | | | | |
| 0-10 years | | 529 | 178(33.65) | | 351(66.35) | | | | |  | | |  | | | | | |
| 10-20 years | | 88 | 88(100) | | 0(0) | | | | |  | | |  | | | | | |
| More than 20 years | | 199 | 51(25.63) | | 148(74.37) | | | | |  | | |  | | | | | |
| **Job title** | |  |  | |  | | | | | 1.3235 | | | 0.5159 | | | | | |
| Junior | | 541 | 155(28.65) | | 386(71.35) | | | | |  | | |  | | | | | |
| Intermediate | | 367 | 95(25.89) | | 272(74.11) | | | | |  | | |  | | | | | |
| senior | | 200 | 60(30) | | 140(70) | | | | |  | | |  | | | | | |
| **Marital status** | |  |  | |  | | | | | 6.0601 | | | 0.0483 | | | | | |
| married | | 725 | 203(28) | | 522(72) | | | | |  | | |  | | | | | |
| unmarried | | 287 | 103(35.89) | | 184(64.11) | | | | |  | | |  | | | | | |
| other | | 36 | 11(30.56) | | 25(69.44) | | | | |  | | |  | | | | | |
| **Educational background** | | |  | |  | | | | | 0.4585 | | | 0.7951 | | | | | |
| Postgraduate and above | | 103 | 34(33.01) | | 69(66.99) | | | | |  | | |  | | | | | |
| Bachelor's degree (undergraduate) | | 762 | 227(29.79) | | 535(70.21) | | | | |  | | |  | | | | | |
| College and below | | 183 | 56(30.6) | | 127(69.4) | | | | |  | | |  | | | | | |
